# Divergent roles of serum CXCL9 as a biomarker in ILD and COPD: a comparative study

**DOI:** 10.3389/fphar.2026.1730688

**Published:** 2026-02-03

**Authors:** Chengsheng Yin, Xin Kang, Yuan Zhang, Jiacui Song, Takehiro Hasegawa, Ling Yao, Yang Hu, Huiping Li

**Affiliations:** 1 Department of Respiratory Medicine, Shanghai Pulmonary Hospital, Tongji University School of Medicine, Shanghai, China; 2 Department of Pulmonary and Critical Care Medicine, Peking Union Medical College Hospital, Chinese Academy of Medical Sciences and Peking Union Medical College, Beijing, China; 3 Research and Development Division, Sysmex R&D Centre Europe GmbH, Hamburg, Germany; 4 Application Support, Global Management, Sysmex Corporation, Kobe, Japan

**Keywords:** chronic obstructive pulmonary disease, CXCL9, immune phenotyping, interstitial lung disease, pulmonary biomarkers, type 1 inflammation

## Abstract

**Background:**

The chemokine CXCL9, induced by interferon-γ (IFN-γ), is a hallmark of type 1 (T1) inflammation. Its role in chronic respiratory diseases remains unclear, with conflicting evidence suggesting it may reflect steroid-responsive inflammation in interstitial lung disease (ILD) but correlate with worse function in chronic obstructive pulmonary disease (COPD).

**Methods:**

Serum levels of CXCL9, KL-6, SP-A, and CRP were measured in 83 ILD patients (with paired samples before and after treatment), 94 COPD patients, and 100 healthy controls (50 smokers and 50 non-smokers). Lung function and biomarker correlations were analyzed, and unsupervised cluster analysis was used to explore inflammatory phenotypes.

**Results:**

CXCL9 levels were markedly elevated in both ILD (median: 57.4 pg/mL) and COPD (70.1 pg/mL) compared to healthy smokers (32.5 pg/mL) and non-smokers (37.0 pg/mL). In COPD, CXCL9 correlated with KL-6 (r = 0.459) and SP-A (r = 0.274), indicating neutrophilic inflammation and epithelial injury. In ILD, higher baseline CXCL9 levels predicted subsequent improvement in lung function and declined following treatment. Cluster analysis revealed divergent CXCL9 and KL-6 trajectories linked to disease outcomes, underscoring their value as dynamic, disease-specific biomarkers.

**Conclusion:**

CXCL9 levels correlate with divergent roles in ILD and COPD. It may serve as a prognostic marker, identifying treatable inflammation in ILD and inflammatory burden in COPD.

## Background

CXCL9 is a small chemokine (∼12 kDa, 103 amino acids) primarily induced by interferon-gamma (IFN-γ) and secreted by macrophages, epithelial cells, endothelial cells, and astrocytes. In the respiratory tract, its key cellular sources include airway macrophages, bronchial epithelial cells, and pulmonary endothelial cells—cell types tightly linked to the pathophysiology of interstitial and obstructive lung diseases (Hasegawa et al., 2023; Nie et al., 2008). Its receptor, CXCR3, is expressed on Th1 cells, CD8^+^ T cells, natural killer (NK) cells, and innate lymphoid cells. CXCL9 orchestrates classical type 1 (T1) immune responses via the CXCL9–CXCR3 axis and shares functional similarities and receptor usage with CXCL10 and CXCL11—other IFN-γ–inducible chemokines that promote Th1 cell recruitment and cytotoxic immune activation.

Experimental models show that knockout of the CXCR3 gene substantially alleviates cigarette smoke–induced lung inflammation, reduces CD8^+^ T cell infiltration, and downregulates IFN-γ and CXCL9 expression, highlighting the pivotal role of this axis in pulmonary immune injury (Metzemaekers et al., 2017). Clinically, CXCL9 is upregulated in several inflammatory lung diseases, including asthma, COVID-19, and interstitial lung disease (ILD). Together, these findings suggest that type 1 (T1) inflammation is pivotal to the pathophysiology of both acute and chronic inflammatory respiratory disorders (Mifsud et al., 2021), with CXCL9 emerging as a core marker of this pathway. In COVID-19 patients, elevated CXCL9 levels are closely linked to severe outcomes such as acute respiratory distress syndrome (ARDS) and renal failure (Hasegawa et al., 2022; Hasegawa et al., 2021).

Marked CXCL9 elevation has also been observed in Stevens–Johnson syndrome and toxic epidermal necrolysis—both CD8^+^ T cell-driven diseases—further supporting its role in Th1-skewed inflammation (Mifsud et al., 2021). In asthma patients, serum CXCL9 concentrations are notably elevated compared to those in healthy controls, underscoring CXCL9’s specificity for T1 inflammation. Studies indicate that CXCL9 levels increase with age in asthma patients, whereas type 2 (T2) inflammatory cytokines tend to decline, implying a possible immunological overlap with chronic obstructive pulmonary disease (COPD) (Hasegawa et al., 2019).

Although T1 inflammation is pivotal to the pathophysiology of acute and chronic inflammatory respiratory diseases, clinical assessment currently faces challenges: traditional systemic inflammatory markers (such as C-reactive protein, CRP) have “diagnostic blind spots” and often fail to identify persistent local inflammation in the lungs of COPD and ILD patients. Meanwhile, fibrosis markers (such as KL-6) mainly reflect accumulated damage and lack sensitivity to early immune activity. Consequently, a biomarker that can sensitively and dynamically reflect immune activity status before irreversible remodeling occurs is urgently needed.

Previous studies, including our own, suggest that CXCL9 may fill this gap, yet it exhibits distinct inflammatory functions in different disease contexts. In our work on COPD, we identified CXCL9 as an independent T1 indicator distinct from eosinophilic T2 inflammation; its elevation correlated with exacerbated neutrophilic inflammation, elevated lung injury markers (such as LDH and KL-6), and declined lung function (%FEV1), suggesting its primary involvement in pathogenic injury (Bafadhel et al., 2011; Chengsheng et al., 2025). However, in ILD (particularly autoimmune-related subtypes), high baseline CXCL9 levels were associated with a favorable response to corticosteroids or immunosuppressive therapy, suggesting this may be a reversible, hormone-sensitive “Treatable Trait” (Kameda et al., 2020). These distinct clinical associations imply that the same T1 immune signal may exert disease-specific immunopathological roles within the different microenvironments of chronic respiratory diseases.

To unravel the clinical utility of this potential mechanism, we employed a combined cross-sectional and longitudinal design to directly compare serum CXCL9 characteristics in ILD and COPD patients. We assessed the correlation of CXCL9 with established pulmonary biomarkers and lung function parameters, and identified specific inflammatory subtypes masked by CRP through cluster analysis. This study aims to determine whether CXCL9 represents a shared inflammatory feature in interstitial and obstructive lung diseases or possesses disease-specific clinical significance, thereby establishing its use as a stratification tool for disease assessment in real-world clinical cohorts.

## Methods

### Patient selection

A total of 206 ILD patients admitted to Shanghai Pulmonary Hospital were screened. After excluding 17 patients with incomplete records, 102 with insufficient respiratory follow-up, and 4 with comorbid non-autoimmune inflammatory conditions, 83 ILD patients were included. Among 100 patients initially enrolled with COPD, 2 were excluded due to intervals exceeding 3 months between serum collection and lung function testing, and 4 due to comorbid infections or inflammatory diseases. In total, 94 COPD patients were analyzed.

Healthy controls included 50 healthy smokers (defined as individuals aged 18–65 years, smoking ≥5 cigarettes/day for ≥10 years, with no respiratory symptoms, normal lung function [post-bronchodilator FEV1/FVC ≥ 0.7, FEV1%pred ≥ 80%], and no history of chronic diseases). The healthy smoker group was exclusively male to match the male-predominant distribution observed in the COPD cohort (85.4%) and reflecting the low prevalence of smoking among females in the study population. Additionally, 50 healthy non-smokers (no smoking history, matching age/sex distribution with healthy smokers, and same exclusion criteria for respiratory/chronic diseases) were included.

The study adhered to the Declaration of Helsinki and was approved by the institutional ethics committee (K17-016). Informed consent was obtained in line with national ethical guidelines. Study details were posted on the hospital website, allowing participants to opt out; thus, written informed consent was waived.

### Study design and data collection

Serum levels of CXCL9, surfactant protein A (SP-A), and Krebs von den Lungen-6 (KL-6) were measured using a fully automatic immunoanalyzer (HISCL™-5000; Sysmex Corp., Hyogo, Japan). Assays followed the chemiluminescent enzyme immunoassay (CLEIA) principle using manufacturer-provided reagents. Briefly, the CXCL9 assay employed a two-step sandwich method. The lower limit of detection (LOD) was 1.0 pg/mL, and the intra- and inter-assay coefficients of variation (CVs) were less than 5.0%. For KL-6 and SP-A, measurement ranges were 10–6,000 U/mL and 1–1,000 ng/mL, respectively. Quality control used high and low control sera for each run to ensure reproducibility. C-reactive protein (CRP) levels, hematological results, and biochemical test results were obtained from medical records. Fibrosis and emphysema extent were quantified using AI-based image analysis (Wu et al., 2022).

### Definitions

ILD was diagnosed based on multidisciplinary consensus and the 2022 international guidelines, including IPF, idiopathic nonspecific interstitial pneumonia, CTD-ILD, chronic hypersensitivity pneumonitis, and unclassifiable ILD (Raghu et al., 2022). COPD was diagnosed per GOLD criteria, defined as post-bronchodilator FEV_1_/FVC < 0.7. Regarding disease progression in ILD patients, we adopted a 10% absolute change in %FVC as the classification cutoff. According to ATS/ERS guidelines and relevant literature, this magnitude of change is generally considered the minimal clinically important difference (MCID), particularly when assessing short-term disease progression.

### Statistical analysis

Due to non-normal distributions, data are presented as medians with interquartile ranges (IQR). Statistical analyses were performed in R (version 4.0.3) using the Mann–Whitney U test, Fisher’s exact test, and Steel–Dwass test. The Wilcoxon test was used to assess longitudinal biomarker changes. For cluster analysis, unsupervised hierarchical clustering was performed using Cluster 3.0 (University of Tokyo Human Genome Center). Prior to clustering, biomarker data were normalized using z-score transformation (mean = 0, standard deviation = 1) to ensure comparability across variables. Clustering was executed using the Euclidean distance metric and the complete linkage algorithm.

## Results

### Demographics of the patient and biomarker levels

This retrospective cross-sectional study examined serum CXCL9 levels in ILD and COPD. For ILD, we analyzed serum CXCL9 and other respiratory biomarkers at two time points to investigate longitudinal changes and their relationship with disease prognosis.

Table 1 presents patient demographics: 83 with ILD, 94 with COPD, 50 healthy smokers, and 50 healthy non-smokers. Median ages in the ILD and COPD groups were 62.0 and 68.0 years, respectively, markedly higher than the healthy control group (median age: 42.5 years). The cohort was predominantly male, with 85.4% of COPD patients and 100% of healthy smokers being male.

**TABLE 1:** Demographics and clinical characteristics.

Variables	ILD	COPD	Healthy smokers	Healthy non-smokers
Age years, median (IQR)	62.0 (53.5–68.0)	68.0 (61.8–73.3)	36 (29.3–44.8)	42.5 (38.0–47.8)
Sex, male, n (%)	52 (62.7)	70 (85.4)	47 (100.0)	33 (64.7)
Smokers	48	66	47	0
FVC% pred, median (IQR)	65.3 (50.2–80.7)	70.4 (57.2–83.0)	NA	NA
FEV1% pred, median (IQR)	70.0 (55.0–82.2)	50.6 (36.8–67.0)	NA	NA
FEV1/FVC% pred, median (IQR)	84.8 (79.7–89.5)	60.1 (47.2–66.8)	NA	NA
DLco, median (IQR)	54.2 (45.0–66.6)	72.8 (58.7–87.8)	NA	NA
SpO2, median (IQR)	96.5 (94.9–97.3)	96.3 (94.8–97.5)	NA	NA
PaO2, median (IQR)	82.0 (74.8–93.3)	83.0 (73.0–88.5)	NA	NA
PaCO2, median (IQR)	39.2 (36.8–41.6)	41.8 (39.3–45.6)	NA	NA
A-aDO2, median (IQR)	23.0 (11.5–34.6)	NA	NA	NA
Improved group, n (%)	25 (30.1)	NA	NA	NA
Unchanged group, n (%)	43 (51.8)	NA	NA	NA
Declined group, n (%)	15 (18.1)	NA	NA	NA
ILD classification				
CTD-ILD, n (%)	15 (18.1)	NA	NA	NA
IPAF, n (%)	1 (1.2)	NA	NA	NA
IPF, n (%)	27 (32.5)	NA	NA	NA
PAP, n (%)	5 (6.0)	NA	NA	NA
ILD, n (%)	35 (42.2)	NA	NA	NA
COPD, n (%)	NA	78 (97.5)	NA	NA
COPD-RA, n (%)	NA	2 (2.5)	NA	NA
GOLD I	NA	8 (10.0)	NA	NA
GOLD II	NA	32 (40.0)	NA	NA
GOLD III	NA	19 (23.8)	NA	NA
GOLD IV	NA	18 (22.5)	NA	NA
Medication				
Before pirfenidone, n (%)	2 (2.4)	NA	NA	NA
After pirfenidone, n (%)	5 (6.0)	NA	NA	NA
Before steroids, n (%)	47 (56.6)	NA	NA	NA
After steroids, n (%)	43 (51.8)	NA	NA	NA
ICS, n (%)	0 (0.0)	30 (37.5)	NA	NA
LABA, n (%)	0 (0.0)	3 (3.8)	NA	NA
LAMA, n (%)	0 (0.0)	35 (43.8)	NA	NA
SABA, n (%)	0 (0.0)	11 (13.8)	NA	NA
KL-6 U/mL, median (IQR)	1,105.0 (564.0–1951.5)	232.0 (157.0–288.0)	162.0 (123.5–196.8)	151.5 (120.3–177.5)
SP-A ng/mL, median (IQR)	60.0 (44.3–93.0)	35.8 (28.2–46.7)	21.7 (15.0–31.1)	25.4 (20.1–33.2)
CRP mg/dL, median (IQR)	5.0 (2.5–10.2)	4.8 (3.1–8.55)	NA	NA
LDH U/L, median (IQR)	210.0 (176.8–250.0)	171.0 (142.0–185.0)	NA	NA

Data are presented as median, (IQR), n (%), or n/N (%), where N is the total number of patients with available data. PAP: Pulmonary alveolar proteinosis CTD-ILD: Connective tissue disease-associated interstitial lung disease; COPD-RA: COPD, patients with co-morbid Rheumatoid Arthritis.

The median forced vital capacity percent predicted (%FVC) for ILD and COPD was 65.3% and 70.4%, respectively. Correspondingly, the median predicted forced expiratory volume in 1 s (%FEV1) was 70.0% and 50.6%, respectively. Within the COPD group, patient classifications based on Global Initiative for Chronic Obstructive Lung Disease (GOLD) criteria were: 10.0% GOLD I, 40.0% GOLD II, 23.8% GOLD III, and 22.5% GOLD IV. ILD participants with a decrease of ≥10% in %FVC after 4–9 months were classified into the’declined’group, while those with an increase of ≥10% were classified into the’improved’group. This resulted in 15 cases in the declined group and 25 cases in the improved group.

The ILD group included 15 (18.1%) patients with CTD-ILD, 1 (1.2%) with interstitial pneumonia with autoimmune features (IPAF), 27 (32.5%) with IPF, 5 (6%) with PAP (Pulmonary Alveolar Proteinosis), and 35 (42.2%) with unclassified ILD. Two patients (2.4%) received antifibrotic treatment, and 47 (56.6%) received steroids before the first sample collection. Two patients also had rheumatoid arthritis (RA), and 30 (37.5%) received inhaled corticosteroids. Median serum Krebs von den Lungen-6 (KL-6) levels in ILD patients, COPD patients, healthy smokers, and healthy non-smokers were 1,105.0, 232.0, 162.0, and 1,51.5 U/mL, respectively. Median serum surfactant protein A (SP-A) levels were 60.0, 35.8, 21.7, and 25.4 ng/mL, respectively.

**FIGURE 1:**
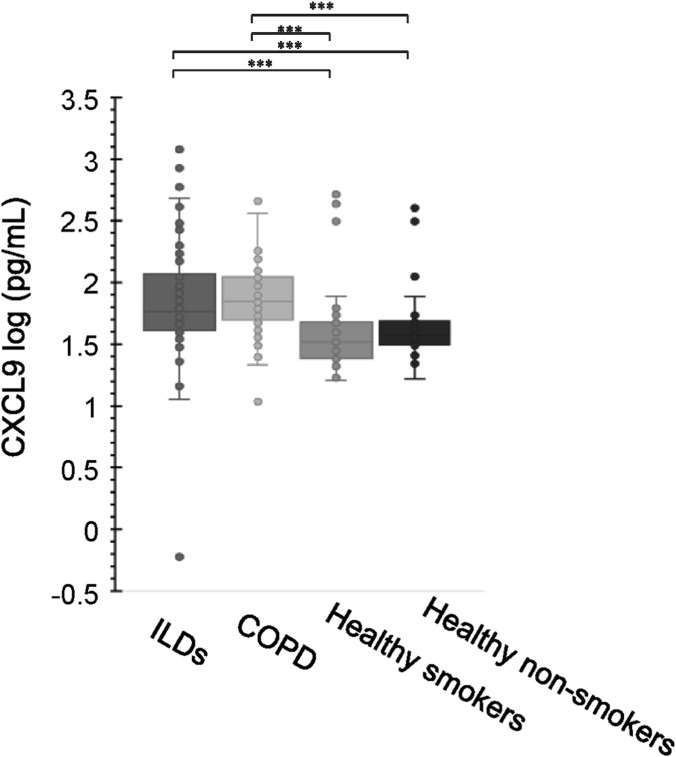
The distribution of CXCL9 levels in ILD patients, COPD patients, healthy smokers, and healthy non-smokers. The statistical significances between the clusters were calculated using the Steel–Dwass test. *P < 0.001.

Median serum CXCL9 concentrations in ILD patients, COPD patients, healthy smokers, and healthy non-smokers were 57.4, 70.1, 32.5, and 37.0 pg/mL, respectively (Figure 1). Serum CXCL9 levels were significantly higher in individuals with ILD and COPD compared to healthy smokers and non-smokers. However, no significant differences were observed between ILD and COPD or between healthy smokers and non-smokers. We retained the two healthy cohorts to explicitly exclude smoking as a confounding factor—this design ensures that elevated CXCL9 in diseased groups is attributed to ILD/COPD pathology rather than smoking itself, even if smoking does not independently alter CXCL9 levels in healthy individuals.

### Association between serum CXCL9 concentrations and the pathophysiological features of respiratory diseases

To investigate the association between serum CXCL9 concentrations and the pathophysiological characteristics of ILD and COPD, we analyzed correlations between CXCL9 levels and several parameters. In ILD patients, CXCL9 levels exhibited a weak, significant negative correlation with %FVC on the first day of sample collection and a positive correlation with the change in %FVC from the first day. Conversely, in COPD patients, CXCL9 levels were significantly associated with KL-6 and SP-A levels. The correlation coefficients for SP-A and KL-6 were 0.459 and 0.274, respectively. Meanwhile, in ILD patients, CXCL9 levels were also negatively correlated with both %FEV1 and lymphocyte count, though the correlation coefficients were weak (Table 2).

**TABLE 2:** Correlations among pathophysiological parameters and CXCL9 levels in patients.

Variables	ILD		COPD	
	rS	P	rS	P
Age	0.204	0.065	0.302	0.058
SP-A	0.060	0.588	0.459	<0.001
KL-6	−0.063	0.569	0.274	0.013
Delta SP-A	−0.133	0.254	0.016	0.886
Delta KL-6	−0.099	0.398	−0.063	0.576
%FVC	−0.218	0.048	−0.259	0.019
%FEV1	−0.150	0.176	−0.144	0.269
FEV1%	−0.043	0.699	−0.159	0.155
DLco	−0.090	0.429	−0.115	0.307
SpO2	−0.032	0.778	0.023	0.837
PaO2	−0.064	0.570	−0.008	0.941
PaCO2	0.019	0.865	−0.122	0.276
A-aDO2	0.055	0.627	−0.153	0.169
FVC relative change	0.344	0.001	−0.065	0.563
FVC absolute change	0.334	0.002	0.157	0.160
Fibrosis score	−0.002	0.985	−0.264	0.017
Emphysema rate	−0.092	0.407	0.146	0.193
Hb	−0.177	0.116	0.225	0.042
RBC	−0.070	0.538	−0.007	0.947
WBC	−0.054	0.635	0.055	0.624
Neutrophil %	0.105	0.352	−0.268	0.015
Lymphocyte %	−0.136	0.229	0.045	0.690
Monocyte %	0.051	0.654	0.223	0.044
Eosinophils %	0.176	0.119	0.063	0.571
Basophil %	−0.004	0.970	0.069	0.535
Neutrophil count	−0.025	0.825	−0.073	0.516
Lymphocyte count	−0.224	0.046	0.308	0.008
Monocyte count	0.032	0.777	0.320	0.005
Eosinophils count	0.174	0.123	0.302	0.058
Basophil count	0.003	0.978	0.459	<0.001
Platelet count	0.058	0.611	0.274	0.013
Haematocrit	−0.122	0.280	0.016	0.886
LDH	−0.011	0.926	−0.063	0.576
CRP	0.239	0.030	−0.259	0.019

Significant correlations are indicated. The correlation coefficients were generated by Spearman’s correlation analysis. Results were considered significant at P < 0.05.

### COPD and levels of serum inflammatory markers

**FIGURE 2:**
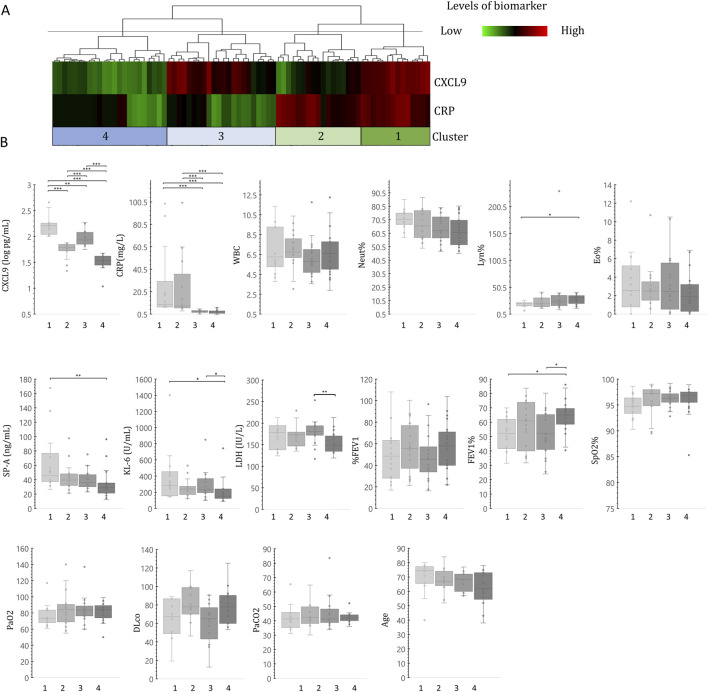
Cluster analysis of serum CXCL9 and KL-6 levels in COPD patients. Unsupervised hierarchical clustering analysis of serum CXCL9 and KL-6 levels **(A)**. The cluster analysis was performed using a complete linkage approach based on City-Block distance. Comparison of patient characteristics in the four identified clusters **(B)**. Each cluster is indicated on the x-axis. The statistical significances between the clusters were calculated using the Steel–Dwass test. P < 0.05, **P < 0.01, ***P < 0.001.

In COPD patients, CXCL9 showed a weak negative correlation with CRP (rS = −0.259), a marker related to COPD inflammation. We investigated differences in inflammatory pathophysiology between CRP and CXCL9 using unsupervised hierarchical cluster analysis. Patients with COPD were categorized into four groups based on serum CRP and CXCL9 levels: Cluster 1, high CXCL9 and CRP; Cluster 2, high CRP and low CXCL9; Cluster 3, high CXCL9 and normal CRP; and Cluster 4, low CXCL9 and CRP levels. Analysis revealed no significant difference in CRP levels between Clusters 1 and 2 (Figures 2A,B).

Levels of the ILD marker KL-6 were significantly higher in Clusters 1 and 3, where CXCL9 levels were high. In contrast, KL-6 levels were low in Cluster 2, where CRP levels were high. SP-A levels in Cluster 1 were also higher than those in Cluster 4. Additionally, LDH levels were increased in Cluster 3. Moreover, FEV1% was significantly lower in Clusters 1 and 3 than in Cluster 4 (1 vs. 4 and 3 vs. 4, *P =* 0.026 and *P* = 0.045, respectively). Cluster analysis indicated that patients in Clusters 1 and 3 displayed heightened severity compared to those in Clusters 2 and 4, even though CRP levels were low in Cluster 3. This tendency was also observed in Clusters 1 and 3 for oxygen saturation (SpO2), DLco, and PaO2 (Figure 2B).

### ILD and biomarker levels

We then explored the relationship between CXCL9 levels and the change in %FVC during the 4–9 months following the initial sample collection for ILD. In this population, %FVC and %FEV1 were significantly higher in the declined group than in the improved group. Values in the improved group were also significantly lower than those in the unchanged group.

**FIGURE 3:**
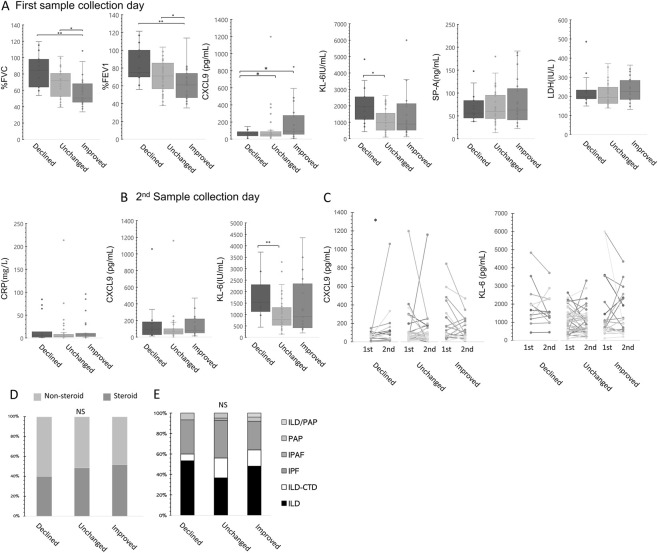
Respiratory function test results and serum biomarker levels in ILD patients. Respiratory function test results and serum biomarker levels in ILD patients on the first sample collection day **(A)**. Patients were classified into declined, unchanged, and improved groups based on the %FVC change. Serum CXCL9 and KL-6 levels on the second sample collection day **(B)**. Changes in CXCL9 and KL-6 levels between the first and second sample collection days **(C)**. Steroid treatment ratios in the different groups **(D)**. Proportions of ILD disease types **(E)**. The statistical significances between the clusters were calculated using the Steel–Dwass test, Wilcoxon test, or Fisher’s exact test. P < 0.05, **P < 0.01, ***P < 0.001.

CXCL9 levels in the improved group on the first sampling day were markedly higher than those in the declined and unchanged groups. Additionally, KL-6 levels in the declining group on the first sampling day were significantly higher than those in the unchanged group. However, no significant variations were found in SP-A, LDH, and CRP levels among the improved, unchanged, and declined groups (Figure 3A). Nonetheless, multiple regression analysis revealed a significant association between CXCL9 levels and changes in respiratory function after adjusting for age, sex, and steroid use (Supplementary Table S1).

Elevated CXCL9 levels in the improved group disappeared by the second sample collection day, whereas the increase in KL-6 levels in the declined group was sustained (Figure 3B). Comparing serum levels on the first and second sample collection days, CXCL9 levels demonstrated a significant increase in the declining group (P = 0.048). Moreover, although a significant reduction in CXCL9 levels in the improved group was not detected using the Wilcoxon test, there was a decrease in cases where initial CXCL9 levels were > 200 pg/mL on the second day. Conversely, KL-6 levels did not differ among the three groups (Figure 3C). Regarding baseline characteristics, the proportion of patients receiving corticosteroid treatment did not differ significantly among the Improved, Unchanged, and Declined groups (Figure 3D). Furthermore, the distribution of ILD subtypes (IPF, CTD-ILD, etc.) was comparable across these outcome groups, suggesting that the prognostic value of CXCL9 is not driven by a specific disease subtype (Figure 3E).

### Change of biomarker levels and outcome of disease in ILD

**FIGURE 4:**
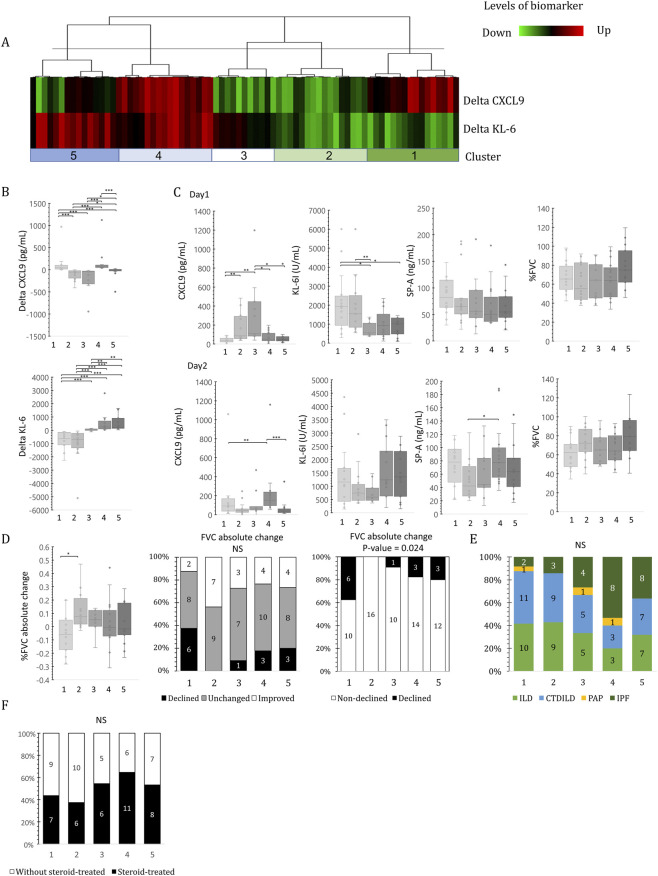
Cluster analysis of serum CXCL9 and KL-6 level changes in ILD patients. Unsupervised hierarchical clustering analysis of serum CXCL9 and KL-6 level changes between the first and second sample collection days **(A)**. The cluster analysis was performed using a complete linkage approach based on correlation distance. Changes in CXCL9 and KL-6 levels **(B)**. Comparison of patient characteristics in the five identified clusters in the first (Upper) and second (Lower) sampling days **(C)**. Changes in %FVC **(D)**. Proportions of ILD disease types **(E)**. Ratio of steroid-treated patients (filled boxes) among the clusters **(F)**. Each cluster is indicated on the x-axis. The statistical significances between the clusters were calculated using the Steel–Dwass test or Fisher’s exact test. *P < 0.05, **P < 0.01, ***P < 0.001.

CXCL9 and KL-6 levels showed a significant relationship with respiratory outcomes. We investigated the relationship between both markers and changes in respiratory function using unsupervised hierarchical cluster analysis (Figure 4A). ILD cases were categorized into five distinct groups according to changes in KL-6 and CXCL9 levels between the first and second sample collection days: Cluster 1, marked by CXCL9-induced KL-6 reduction; Cluster 2, displaying reductions in both KL-6 and CXCL9; Cluster 3, indicating only CXCL9 reduction; Cluster 4, characterized by both CXCL9 and KL-6 induction; and Cluster 5, denoting KL-6 induction (Figure 4A). The magnitude of biomarker changes varied distinctly across clusters; specifically, Cluster 1 exhibited a substantial increase in KL-6 alongside rising CXCL9, whereas Cluster 2 showed concurrent reductions in both markers (Figure 4B).

On the first day of sample collection, CXCL9 levels were significantly higher in Clusters 2 and 3, whereas KL-6 levels were higher in Clusters 1 and 2. On the second day, median CXCL9 levels in Clusters 2 and 3 decreased by 45.6 and 70.4 pg/mL, respectively. In contrast, in Clusters 1 and 4, it increased by 91.8 and 157.6 pg/mL, respectively (Figure 4C). On the second sample collection day, median KL-6 levels decreased by 1,156.5 and 737.5 U/mL in Clusters 1 and 2, respectively. In Clusters 4 and 5, these levels increased to 1,240 and 1,353 U/mL, respectively. Furthermore, median SP-A levels in Cluster 4 were the lowest on the first sample collection day but were the highest among the clusters on the second day. Despite the absence of a significant difference in %FVC among clusters on either day, the %FVC of Cluster 2, which initially demonstrated the lowest value, experienced a median improvement of 7.4% on the second collection. Significant differences were observed in the change in %FVC between Clusters 1 and 2 (P = 0.0134). However, Cluster 2 did not include any patients classified as having a decline in %FVC; instead, 44% of patients in this cluster were classified into the improvement group (Figure 4D). This result remained consistent when relative %FVC change was employed for classification. Nevertheless, no statistically significant bias was observed; half of the patients in Cluster 4, who exhibited reduced levels of CXCL9 and KL-6, were indicative of IPF. In contrast, disease types in Clusters 1 and 2 were comparable (Figure 4E). Notably, corticosteroid usage was distributed across all clusters, with no statistically significant enrichment in any specific phenotypic group (Figure 4F), indicating that these inflammatory clusters reflect biological heterogeneity beyond simple treatment effects.

## Discussion

We systematically analyzed the expression of CXCL9, a T1 inflammation-related chemokine derived from airway macrophages, bronchial epithelial cells, and pulmonary endothelial cells, in ILD and COPD, investigating its associations with pulmonary function, biomarker levels, and disease severity. We aimed to elucidate common inflammatory pathways and disease-specific mechanisms shared between these two chronic respiratory conditions.

In healthy individuals, serum CXCL9 levels are generally low. Even among those over 60  years old, median levels remained approximately 20.5 pg/mL (IQR: 18.0–22.3), substantially lower than those observed in patients with ILD (57.4 pg/mL) and COPD (70.1 pg/mL) in this study. Although both healthy smokers and nonsmokers exhibited slightly higher CXCL9 levels compared to the 95th percentile reference (39.0 pg/mL) in a Japanese cohort, their values were still markedly lower than those in diseased groups, highlighting CXCL9’s sensitivity as a marker of immune activation.

CXCL9 is a canonical T1 chemokine induced by IFN-γ that recruits Th1 cells, macrophages, and cytotoxic T lymphocytes. Compared to existing markers such as FeNO (fractional exhaled nitric oxide), CXCL9 offers distinct advantages: FeNO is a T2 inflammation-specific marker and cannot reflect T1 inflammation, whereas CXCL9 directly indexes T1 immune activity. Additionally, CXCL9 predicts lung function improvement in ILD (Figure 3C) and correlates with structural injury in COPD (Figure 2B)—features not observed with FeNO, which has limited prognostic value in chronic respiratory diseases. Studies demonstrate it is significantly elevated in the serum and bronchoalveolar lavage fluid (BALF) of patients with CTD-ILD, IPAF, and chronic hypersensitivity pneumonitis (CHP), correlating with disease activity (Nukui et al., 2019). Additionally, CXCL9 is negatively correlated with IL-4, a key T2 cytokine, and is elevated in non-allergic asthma, emphasizing its specificity for non-T2 inflammatory phenotypes. In contrast, KL-6 and SP-A, common ILD biomarkers, are surfactant-associated proteins that leak into circulation due to increased alveolar-capillary permeability (Kuroki et al., 1998; Oyama et al., 1997). These mechanistic differences form the basis for the distinct behaviors of CXCL9 and structural biomarkers in different disease types.

Unsupervised clustering analysis identified a “non-CRP dominant T1 inflammation” subgroup in COPD patients characterized by “high CXCL9, low CRP,” associated with neutrophil infiltration, epithelial damage (such as elevated KL-6 and SP-A), and decreased lung function. Additionally, using paired pre- and post-treatment samples from ILD patients, results indicated that baseline CXCL9 levels were associated with treatment response, potentially aiding in real-time monitoring of ILD treatment efficacy. Unlike previous literature, this study identifies the association between CXCL9 and both ILD and COPD. Through cross-disease comparisons and subgroup analyses, it provides functional insights suitable for classification and evaluation, complementing research on CD206^+^ macrophage-mediated pro-fibrotic mechanisms. Supportive literature indicates that COPD exhibits significant inflammatory heterogeneity, with T1 and T2 inflammation interwoven (Bafadhel et al., 2011), and most biologic agents target the T2 pathway. However, this study shows that CXCL9 is associated with T1 inflammation, linked to epithelial damage in COPD and treatment response in ILD, thus providing insight into the identification of T1 inflammation subgroups. These results highlight the clinical value of T1 inflammation and provide exploratory criteria for patient stratification targeting T1 inflammation.

CXCL9’s role in pathophysiology appears distinct between diseases: in ILD, CXCL9 is associated with reversible, reparative inflammation, recruiting Th1 cells through the CXCR3 axis, with its elevation not accompanied by sustained increases in KL-6, thus correlating with lung function improvement; in COPD, CXCL9 is linked to epithelial damage and airway remodeling, inducing CD8^+^ T cell infiltration, and correlating with lower FEV1%. This observation is based on the integration of clinical data with basic research, such as CXCR3 gene knockout mouse models showing that this axis exacerbates smoke-induced lung damage (Ishikawa et al., 2011a). In COPD, it is known that peripheral CD8^+^ T cells highly express perforin, Toll-like receptors, IFN-γ, and TNF-α—upstream regulators of CXCL9 (Duan et al., 2022; Grumelli et al., 2004; Saetta et al., 2002)—which may explain the observed elevation of CXCL9 in this group. Our prior findings also suggest that CXCL9, as an independent T1 inflammation biomarker distinct from eosinophilic (T2) inflammation in COPD, has potential clinical utility for phenotype analysis and could serve as a candidate target for customized anti-inflammatory therapy. CXCL9 may complement CRP in the evaluation of non-CRP dominant inflammation, particularly in CRP-negative populations (Zhao and Su, 2025).

As a potential biomarker, its dynamic changes with treatment support its role in prognosis—e.g., high baseline CXCL9 can identify ILD patients likely to respond to steroids. Specifically, CXCL9 helps stratify inflammation types, enabling the identification of T1-dominant inflammation subgroups in ILD, thus guiding treatment response evaluation. In COPD, it can aid in distinguishing the non-CRP dominant T1 inflammation phenotype, associated with epithelial damage, supporting the clinical classification of inflammatory heterogeneity. However, the core focus of this study is on exploring the value of CXCL9 as an inflammation stratification and prognostic evaluation biomarker, with all relevant inferences based on current clinical and mechanistic data, rather than direct treatment intervention evidence. Future prospective studies are needed to further validate its applicability in personalized management to avoid extrapolating beyond the current evidence (Celli et al., 2025).

While elevated in both ILD and COPD, CXCL9 shows different associations with clinical biomarkers. In ILD, CXCL9 levels correlate positively with %FVC change. The “improvement” subgroup of patients showed higher baseline CXCL9 levels, which decreased over time, while the “deterioration” subgroup exhibited sustained increases in KL-6 and CXCL9. This suggests that CXCL9 may reflect inflammatory activity and correlate with lung function improvement. Kameda et al. also reported that elevated CXCL9 levels in IPAF/CTD-ILD patients receiving immunosuppressive therapy were associated with better treatment response (Hasegawa et al., 2019). In contrast, in COPD, CXCL9 is positively correlated with KL-6, SP-A, LDH, and CRP (r > 0.3), indicating its association with chronic T1 inflammation, structural damage, and airway remodeling. While some correlations were modest (e.g., r = 0.274), the core conclusions rely on longitudinal associations. Including CXCL9 in COPD clinical assessment may aid in phenotype classification and provide information for precision treatment. Unsupervised clustering further revealed that clusters 1 and 3 exhibited high CXCL9 levels, elevated ILD biomarkers, and decreased FEV1%, though CRP levels were not consistently elevated. This “non-CRP dominant T1 inflammation pattern” resembles patterns observed in severe COVID-19, where elevated CXCL9 along with CRP is associated with ARDS and multi-organ failure (Fermont et al., 2019).

In COPD, CXCL9 is significantly correlated with sub-threshold increases in KL-6 and SP-A but still correlates with poorer lung function, consistent with previous reports suggesting that these biomarkers have prognostic value in COPD (Ishikawa et al., 2011a; Ishikawa et al., 2011b). This suggests that CXCL9-driven inflammation may be associated with epithelial damage even in the absence of obvious biomarker elevation. In ILD, the dynamic co-variation between CXCL9 and KL-6 shows stratification potential. Cluster 2 patients exhibited significant %FVC improvement, while cluster 1 experienced functional decline, suggesting that persistent T1 inflammation may counterbalance epithelial recovery. Cluster 4 showed stable lung function, potentially reflecting early inflammation. Cluster 5 maintained low CXCL9 levels and lacked CTD-ILD/IPAF cases, potentially representing a T2-dominant phenotype (based on indirect evidence, not definitive). Supporting this, Nukui et al. found elevated CCL17 (a T2 chemokine) in chronic bird-related HP (Nukui et al., 2019), and T2 inflammation has been shown to promote myofibroblast differentiation in IPF. Referring to Fahy et al.’s study (Fahy et al., 2025), T1 and T2 inflammation often exhibit mutually exclusive distributions in respiratory diseases; through indirect evidence, low CXCL9 clusters show clinical tendencies associated with T2 inflammation, without the need for direct measurement of T2 biomarkers (Hughes et al., 2023; Ziyatdinov et al., 2025). Clustering analysis is exploratory, and its biological or clinical interpretation is based on existing clinical features.

Although 56.6% of ILD patients received corticosteroid treatment, significant correlations between CXCL9 dynamics and %FVC were still observed (r = 0.334, P = 0.002) (Qureshi et al., 2021). In the corticosteroid-treated ILD subgroup, post-treatment CXCL9 levels correlated positively with %FVC improvement (r = 0.344, P = 0.001). In the untreated subgroup (43.4%), 36% of patients still showed CXCL9 decline accompanied by lung function improvement, consistent with literature suggesting that dynamic changes in inflammatory biomarkers can reflect treatment response (Duan et al., 2022). Although the follow-up period (4–9 months) was shorter than the recommended 12 months, it still captured the dynamic value of CXCL9 as a T1 inflammation biomarker, consistent with Meng et al.’s long-term cohort study, which showed that key inflammatory risk signals could manifest within 6 months after infection (Meng et al., 2024).

This study is a single-center retrospective study with limitations, including incomplete treatment data and the lack of baseline samples before treatment, which may affect the interpretation of CXCL9 as a treatment response biomarker (Pommerolle et al., 2024). We used a 10% absolute change in %FVC as a cutoff, which may have excluded some milder cases. Nevertheless, the core contribution of this study lies in the exploratory clinical relevance, rather than a breakthrough in mechanisms. All mechanistic discussions are based on the integration of existing clinical data and basic research, with a moderate level of evidence (observational data) (Yang et al., 2022).

Additionally, although the distribution of ILD subtypes aligns with the Chinese cohort (Kaul et al., 2021), the proportion of IPF in our study was slightly higher. Similarly, COPD patients in GOLD stages III and IV accounted for 46.3%, which is slightly different from large-scale studies (Yang et al., 2022), possibly reflecting a bias towards more severe cases in our sample. Overall, the sample size of this study is consistent with the typical size for single-center exploratory clinical studies (Raghu et al., 2022), and the subgroup analysis results should be considered as exploratory indications of clinical relevance, not definitive conclusions. The heterogeneity of the ILD group and the proportion of unclassified cases reflect the complexity of ILD diagnosis in domestic clinical practice, consistent with the subtype distribution reported in the Chinese interstitial lung disease cohort. Through subgroup analysis, we found that ILD subtypes and COPD GOLD stages did not significantly interfere with the association between CXCL9 and core indicators. Future research will require longer follow-up in multi-center cohorts to further confirm the clinical application of CXCL9 as a biomarker in chronic respiratory diseases. Specifically, future studies should focus on the dynamic changes of CXCL9 in different center cohorts and validate its actual value in personalized treatment (Cunningham et al., 2022; Jacobson et al., 2025).

In summary, CXCL9 is a T1 inflammation-related chemokine elevated in both ILD and COPD, yet it carries distinct clinical implications. In ILD, CXCL9 is associated with reversible inflammation and lung function recovery, while in COPD, it may represent non-CRP dominant structural inflammation. Longitudinal monitoring of CXCL9, in combination with a multi-biomarker panel, may enhance inflammatory phenotype analysis and provide insights for personalized management strategies in chronic respiratory diseases.

## Data Availability

The raw data supporting the conclusions of this article will be made available by the authors, without undue reservation.
