# Supplementary material for: Divergent roles of serum CXCL9 as a biomarker in ILD and COPD: a comparative study
Source: Front Pharmacol. 2026 Feb 3;17:1730688. doi: 10.3389/fphar.2026.1730688 (PMC12910215; doi:10.3389/fphar.2026.1730688)
Supplement: Supplementary file 1 [file Presentation1.pptx]

## Slide 1
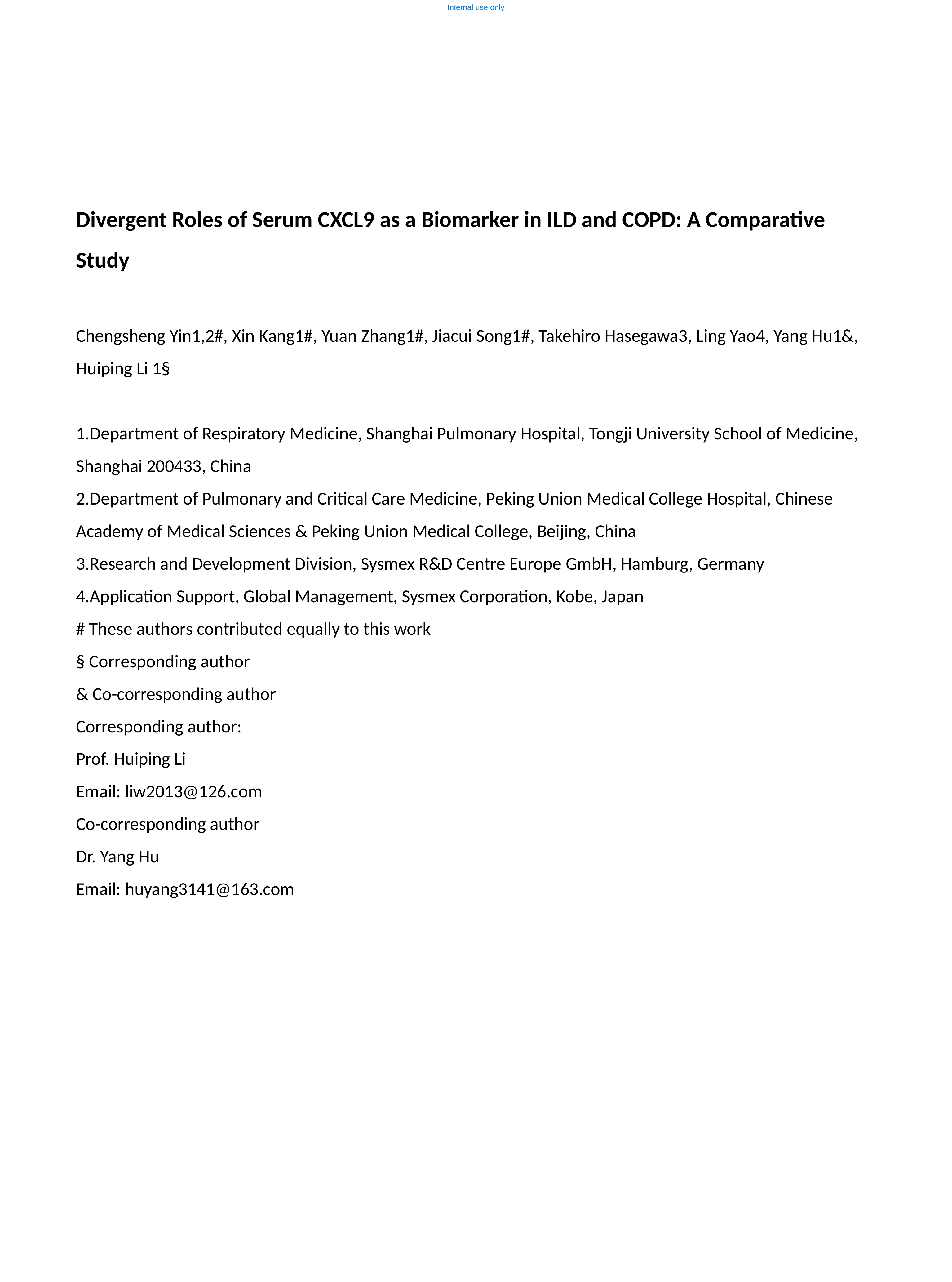

# Divergent Roles of Serum CXCL9 as a Biomarker in ILD and COPD: A Comparative Study Chengsheng Yin1,2#, Xin Kang1#, Yuan Zhang1#, Jiacui Song1#, Takehiro Hasegawa3, Ling Yao4, Yang Hu1&, Huiping Li 1§ 1.Department of Respiratory Medicine, Shanghai Pulmonary Hospital, Tongji University School of Medicine, Shanghai 200433, China 2.Department of Pulmonary and Critical Care Medicine, Peking Union Medical College Hospital, Chinese Academy of Medical Sciences & Peking Union Medical College, Beijing, China 3.Research and Development Division, Sysmex R&D Centre Europe GmbH, Hamburg, Germany 4.Application Support, Global Management, Sysmex Corporation, Kobe, Japan # These authors contributed equally to this work § Corresponding author & Co-corresponding author Corresponding author: Prof. Huiping Li Email: liw2013@126.com Co-corresponding author Dr. Yang Hu Email: huyang3141@163.com

## Slide 2
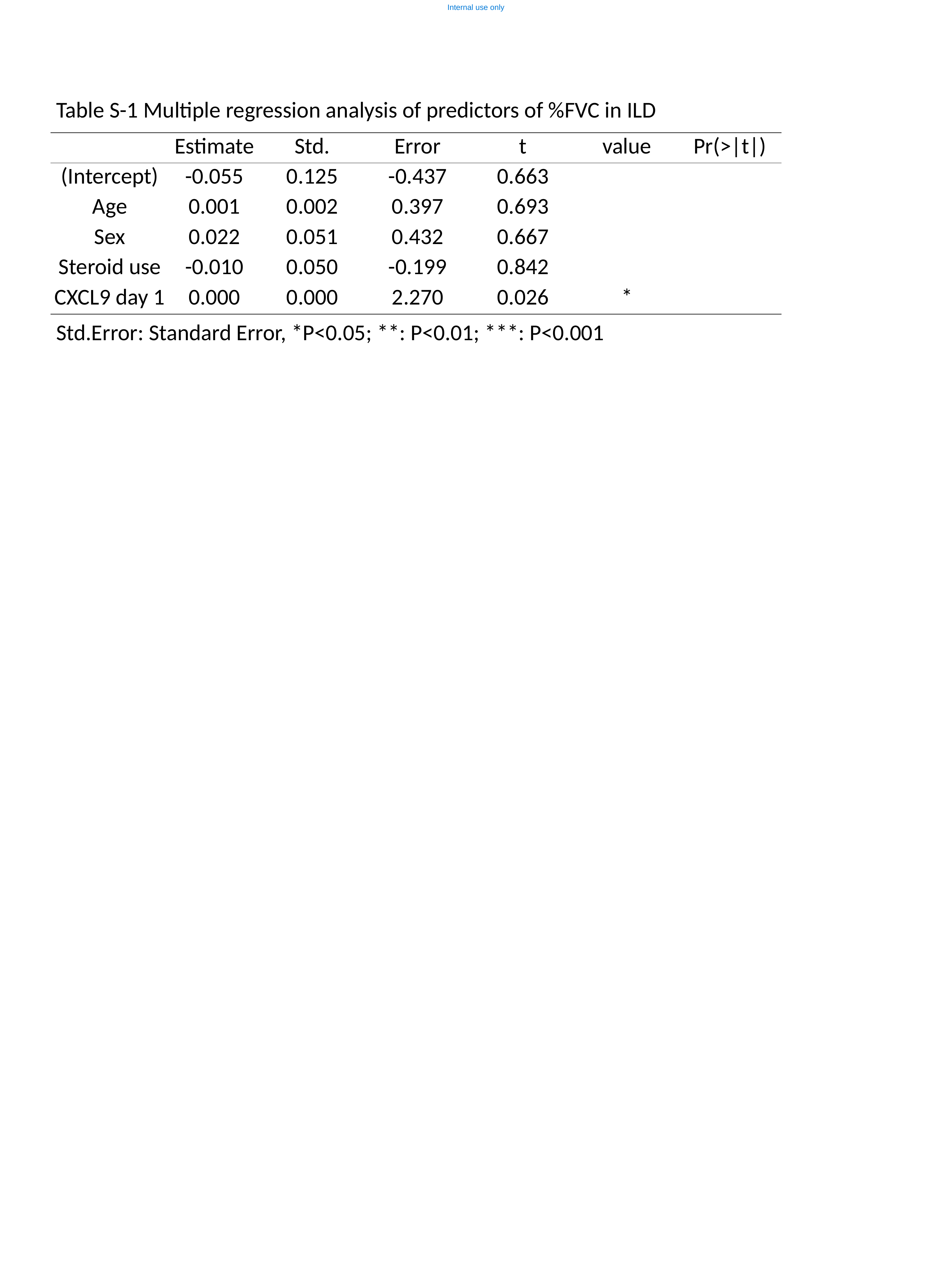

| Table S-1 Multiple regression analysis of predictors of %FVC in ILD | | | | | | |
| --- | --- | --- | --- | --- | --- | --- |
| | Estimate | Std. | Error | t | value | Pr(>|t|) |
| (Intercept) | -0.055 | 0.125 | -0.437 | 0.663 | | |
| Age | 0.001 | 0.002 | 0.397 | 0.693 | | |
| Sex | 0.022 | 0.051 | 0.432 | 0.667 | | |
| Steroid use | -0.010 | 0.050 | -0.199 | 0.842 | | |
| CXCL9 day 1 | 0.000 | 0.000 | 2.270 | 0.026 | \* | |
| Std.Error: Standard Error, \*P<0.05; \*\*: P<0.01; \*\*\*: P<0.001 | | | | | | |
